# IgE-levels to peanut allergen component Ara h 2: relation to peanut symptoms in 8-year-olds

**DOI:** 10.1186/2045-7022-3-S3-P92

**Published:** 2013-07-25

**Authors:** A Asarnoj, G Hedlin, MV Hage, M Wickman

**Affiliations:** 1 Institute of Environmental Medicine, Karolinska Institutet, Stockholm, Sweden; 2 Dept. of Paediatrics, Astrid Lindgren Children’s Hospital, Karolinska University Hospital, Stockholm, Sweden; 3 Clinical Immunology and Allergy Unit, Department of Medicine, Karolinska Institutet and University Hospital, Stockholm, Sweden

## Background

Allergen-specific IgE testing is usually performed with crude peanut extract, and peanut specific IgE-levels are associated with the probability to react at peanut exposure, but cannot predict severity of symptoms. The aim of this study was to investigate levels of IgE to the more clinically relevant peanut allergen component Ara h 2 in children in relation to severity of symptoms.

## Methods

From a birth cohort of 4089 children clinical parameters were obtained through questionnaires. At eight years of age follow up IgE antibody levels between 0.35 and 100 kU/L to peanut extract and peanut allergen components Ara h 1, Ara h 2 and Ara h 3 were measuredusing ImmunoCAP method. Questionnaire data on reactions from peanut was used from both the eight and 12 years of age questionnaire

## Results

Of the 2461 tested children (60% of the original cohort) 195 (7.9%) exhibited IgE to peanut extract above cut off (0.35 kU/L). 185 of those were also analysed for Ara h 1, h2 and h 3 IgE. Peanut symptoms were reported in 70 (82%) of the 85 children with IgE to Ara h 2 at eight years and 75% at 12 years. Type of symptoms at peanut exposure were plotted in relation to peanut Ara h 2 IgE-level. Children with systemic symptoms (GI/urticaria/breathing) to peanut at eight years of age had higher IgE-levels to Ara h 2 (median 27 kU/L) than children with only local symptoms from mouth, eye or nose (median level 0.77 kU/L), p=0.0065. To include Ara h 1 and h 3 in the analysis did not change the results. Four children with rather high Ara h 2 IgE levels, but with no or mild symptoms at eight years reported more severe symptoms at 12 years.

## Conclusion

High IgE-levels to peanut allergen component Ara h 2 seem to correlate with systemic allergic symptoms at peanut exposure.

## Disclosure of interest

None declared.

